# STAT3 inhibitor Stattic and its analogues inhibit STAT3 phosphorylation and modulate cytokine secretion in senescent tumour cells

**DOI:** 10.3892/mmr.2023.12968

**Published:** 2023-02-22

**Authors:** Romana Mikyskova, Olena Sapega, Miroslav Psotka, Ondrej Novotny, Zdeněk Hodny, Sona Balintova, David Malinak, Jana Svobodova, Rudolf Andrys, David Rysanek, Kamil Musilek, Milan Reinis

**Affiliations:** 1 Laboratory of Immunological and Tumour Models, Institute of Molecular Genetics of The Czech Academy of Sciences, 142 20 Prague, Czech Republic; 2 Department of Chemistry, Faculty of Science, University of Hradec Kralove, 500 03 Hradec Kralove, Czech Republic; 3 Laboratory of Genome Integrity, Institute of Molecular Genetics of The Czech Academy of Sciences, 142 20 Prague, Czech Republic

**Keywords:** cellular senescence, docetaxel, signal transducer and activator of transcription 3 inhibition, Stattic, senescence-associated secretory phenotype

## Abstract

Signal transducer and activator of transcription 3 (STAT3) signalling serves an important role in carcinogenesis and cellular senescence, and its inhibition in tumour cells represents an attractive therapeutic target. Premature cellular senescence, a process of permanent proliferative arrest of cells in response to various inducers, such as cytostatic drugs or ionizing radiation, is accompanied by morphological and secretory changes, and by altered susceptibility to chemotherapeutic agents, which can thereby complicate their eradication by cancer therapies. In the present study, the responsiveness of proliferating and docetaxel (DTX)-induced senescent cancer cells to small molecule STAT3 inhibitor Stattic and its analogues was evaluated using tumour cell lines. These agents displayed cytotoxic effects in cell viability assays on both proliferating and senescent murine TRAMP-C2 and TC-1 cells; however, senescent cells were markedly more resistant. Western blot analysis revealed that Stattic and its analogues effectively inhibited constitutive STAT3 phosphorylation in both proliferating and senescent cells. Furthermore, whether the Stattic-derived inhibitor K1836 could affect senescence induction or modulate the phenotype of senescent cells was evaluated. K1836 treatment demonstrated no effect on senescence induction by DTX. However, the K1836 compound significantly modulated secretion of certain cytokines (interleukin-6, growth-regulated oncogene α and monocyte chemoattractant protein-1). In summary, the present study demonstrated differences between proliferating and senescent tumour cells in terms of their susceptibility to STAT3 inhibitors and demonstrated the ability of the new STAT3 inhibitor K1836 to affect the secretion of essential components of the senescence-associated secretory phenotype. The present study may be useful for further development of STAT3 inhibitor-based therapy of cancer or age-related diseases.

## Introduction

Cellular senescence is a state of irreversible proliferation arrest, which results from certain stress inducers, such as chemotherapy, radiotherapy, oncogenic stimuli and replicative exhaustion (1–3). Senescent cells are characterized by typical phenotypic, metabolic and genetic changes, such as flat, large and often multinucleated cells with the presence of multiple vacuoles, increased activity of senescence-associated β-galactosidase, persistent DNA damage response and increased expression of specific cyclin-dependent kinase inhibitors, including p16^ink4a^ and p21^waf1^ (4). Senescent cells produce numerous growth factors, and immunomodulatory and inflammatory cytokines and chemokines, including interleukin (IL)-6, IL-8, IL-1, monocyte chemoattractant protein-1 (MCP-1) and growth-regulated oncogene α (GROα). These factors identify cells with a senescence-associated secretory phenotype (SASP). It has previously been reported that SASP can influence the tissue and tumour microenvironment and further support the development of senescence (5–8).

Signal transducers and activators of transcription (STATs) represent a family of transcription factors, which display signal transduction and transcription regulatory functions (9). The STAT3 signalling pathway serves an important role in the regulation of cell proliferation and produces numerous factors involved in various cellular processes, such as angiogenesis and apoptosis. STAT3 has been recognized as an oncogene, although its effects depend on the particular cellular landscape. Aberrant constitutive activation by STAT3 phosphorylation occurs in a number of human tumours and promotes tumour progression, including promotion of metastasis (10–13). Furthermore, STAT3 pathway activation in different immune cell subtypes is associated with the production of immunosuppressive factors, which can induce myeloid-derived suppressor cells, which serve a major role in tumour promotion (14) and progression (15).

The STAT3 signalling pathway also serves important regulatory roles in cellular senescence. Originally, its involvement was reported in senescence development and maintenance (16,17). However, it has also been reported that STAT3 blockade in *in vitro* murine breast cancer models can induce cellular senescence (18), and that turning off the constitutive activation of STAT3 in certain cancer cell types may induce senescence (19). Therefore, STAT3 pathway activation can be associated both with senescence induction and repression, depending on the cellular landscape and other factors controlling the cell cycle.

Therapeutic STAT3 signalling pathway inhibition using different strategies and targets has been intensively studied. A number of small molecule inhibitors targeting STAT3 phosphorylation have been tested, although none of them has been introduced into clinical practice (20). Stattic (6-nitro-1-benzothiophene 1,1-dioxide) is one of these inhibitors, which is capable of binding to the SH2 domain and inhibiting phosphorylation, dimerization and nuclear translocation of STAT3 (21). Indeed, further modification of the Stattic molecule represents a potential strategy for developing optimized and functional STAT3 pathway inhibitors.

Our previous studies demonstrated that tumour growth of proliferating murine TC-1 HPV-16-associated cancer cells in syngeneic mice was accelerated by co-administration of TC-1 or TRAMP-C2 cancer cells that were made senescent by pre-treatment with the anti-cancer agent docetaxel (DTX) (22). DTX was also successfully used for the induction of senescence in human cell lines (23). These studies suggested that DTX-treated cells can be a useful model for studies focused on senescent cells.

In the present study, a comparative analysis was performed to evaluate the susceptibilities of proliferating and senescent cells, induced by senescence inductor DTX, to the STAT3 inhibitor Stattic, its recently synthesized analogue K1823 (24) and the newly prepared compound K1836. The impacts of the low-toxic compound with phosphorylated-STAT3 (pSTAT3) inhibiting properties, K1836, on tumour cell proliferation, as well as on the SASP of senescent cells were evaluated.

## Materials and methods

### Cell lines

The TC-1 cell line was generated by *in vitro* co-transfection of murine lung C57BL/6 cells with HPV16 E6/E7 and activated human *H-Ras* (G12V) oncogenes (25). In the present study, TC-1 cells were maintained in RPMI 1640 medium (MilliporeSigma) supplemented with 10% foetal calf serum (FCS; Gibco; Thermo Fisher Scientific, Inc.), 2 mM L-glutamine (MilliporeSigma) and antibiotics (100 U/ml gentamicin and 40 µg/ml nystatin; MilliporeSigma) under standard conditions (5% CO_2_, 37°C, 95% relative humidity). The TRAMP-C2 murine prostate cancer cell line was derived from a heterogeneous primary tumour in the prostate of PB-Tag C57BL/6 mice (26). In the present study, TRAMP-C2 cells were maintained in Dulbecco's modified Eagle's medium (DMEM; MilliporeSigma) supplemented with 5% FCS, 5% Nu-Serum IV (BD Biosciences), 5 µg/ml human insulin (MilliporeSigma), dehydroisoandrosterone (10 nM; MilliporeSigma), 100 U/ml gentamicin and 40 µg/ml nystatin in standard conditions (5% CO_2_, 37°C, 95% relative humidity). The human breast adenocarcinoma MDA-MB-231 cell line was cultured in high-glucose (4.5 g/l) DMEM supplemented with 10% FCS, 100 U/ml penicillin and 100 µg/ml streptomycin sulphate (Gibco; Thermo Fisher Scientific, Inc.) under standard conditions (5% CO_2_, 37°C, 95% relative humidity). Mycoplasma testing was performed with a negative result for all cell lines. All cell lines were purchased from the American Type Culture Collection.

### STAT3 inhibitors

**Figure 1.**
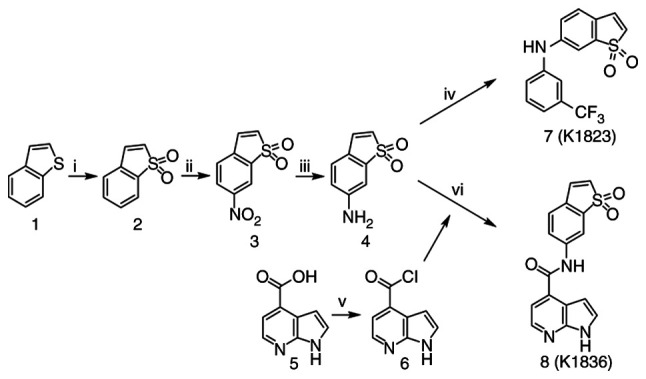
Preparation of compounds K1823 and K1836. Reagents and conditions used were as follows: i) 3-chloroperoxybenzoic acid, DCM, −15°C to reflux, 60 min, 90% yield; ii) HNO_3_, H_2_SO_4_, −15°C to room temperature, 120 min, 73% yield; iii) Fe, NH_4_Cl, H_2_O, MeOH, reflux, 60 min, 72% yield; iv) 1-bromo-3-(trifluoromethyl)benzene, toluene, Cs_2_CO_3_, racemic-2,2′-bis(diphenylphosphino)-1,1′-binaphthalene, Pd(OAc)_2_, 120°C, 3 days, 33% yield; v) (COCl)_2_, DCM, dimethylformamide, −15°C to room temperature, overnight; vi) 6, triethylamine, tetrahydrofuran, DCM, −15°C 15 min then room temperature for 1 h, 10% yield. Chemical formulae are as follows: 1, benzo[*b*]thiophene; 2, benzo[*b*]thiophene-1,1-dioxide; 3,6-nitrobenzo[*b*]thiophene-1,1-dioxide; 4,6-aminobenzo[*b*]thiophene-1,1-dioxide; 5, 1*H*-pyrrolo[2,3-*b*]pyridine-4-carboxylic acid; 6, 1*H*-pyrrolo[2,3-*b*]pyridine-4-carboxylic acid chloride; 7, compound K1823; 8, compound K1836. DCM, dichloromethane.

Stattic was purchased from MedChemExpress. Stattic analogues, K1823 (24) and K1836, were synthesized by the Department of Chemistry, Faculty of Sciences, University of Hradec Kralove (Hradec Kralove, Czech Republic). The compounds' chemical formulae are presented in Fig. 1. Chemical synthesis started from benzo[*b*]thiophene, which was oxidized to corresponding benzo[*b*]thiophene-1,1-dioxide using 3-chloroperoxybenzoic acid as an oxidative agent in dichloromethane. In the next step, C6 was modified by reaction with nitrating mixture HNO_3_/H_2_SO_4_, which produced 6-nitrobenzo[*b*]thiophene-1,1-dioxide. The nitro group was reduced using Fe/NH_4_Cl as a specific reductant in neutral medium (MeOH/H_2_O) to synthesize the necessary amine building block. Compound K1823 was synthesized using a palladium-catalysed Buchwald-Hartwig C-N cross-coupling reaction of 6-amino benzo[*b*]thiophene 1,1-dioxide and 1-bromo-3-(trifluoromethyl)benzene that were stirred in toluene with Cs_2_CO_3_ and catalytic amounts of racemic-2,2′-bis(diphenylphosphino)-1,1′-binaphthalene and Pd(OAc)_2_ for 3 days at 120°C, which yielded the final amine (K1823; 33% yield).

The reactive acyl chloride was prepared overnight from 1*H*-pyrrolo[2,3-*b*]pyridine-4-carboxylic acid using (COCl)_2_ in dichloromethane with a catalytic amount of dimethylformamide (DMF). Prepared 1*H*-pyrrolo[2,3-*b*]pyridine-4-carboxylic acid chloride was treated with 6-aminobenzo[*b*]thiophene 1,1-dioxide in triethylamine (as a base), which generated NH-nucleophiles and produced the final amide (K1836) at 10% yield after two steps. Based on high pressure liquid chromatography analysis with UV detection (λ, 254 nm), the non-calibrated purity of the K1823 and K1836 final products was >95%. Nuclear magnetic resonance spectra of the K1823 and K1836 compounds are presented in Fig. S1, Fig. S2, Fig. S3, Fig. S4.

### Antibodies

The antibodies were used for western blotting were as follows: Rabbit monoclonal pSTAT3 (Y705; 1:2,000; cat. no. 9145S; Cell Signaling Technology, Inc.), mouse monoclonal STAT3 (1:1,000; cat. no. 9139S; Cell Signaling Technology, Inc.), rabbit monoclonal GAPDH (1:10,000; cat. no. 2118S; Cell Signaling Technology, Inc.). The secondary antibodies used were as follows: Goat IgG-HRP anti-rabbit (1:2,000; cat. no. 7074S; Cell Signaling Technology, Inc.) and goat IgG-HRP anti-mouse (1:10,000; cat. no. ab6789; Abcam).

### Senescence induction

To induce senescence in tumour cells, either TC-1 or TRAMP-C2 cells were cultured in fresh medium for 24 h, 7.5 µM DTX (Actavis LLC) was then added to the culture. The dose of DTX was selected as the dose able to induce senescence but not apoptosis based on previous studies (7,22). Tumour cells were cultured in the medium containing DTX for 4 days. Senescence in tumour cells was confirmed by assessment of senescence-associated β-galactosidase (SA-β-gal) activity using a SA-β-gal Staining Kit (Cell Signaling Technology, Inc.) according to the manufacturer's instructions. Images of cell cultures were captured using an inverted Leica DMI 8 light microscope (Leica Microsystems GmbH). In the experiments comparing proliferating and senescent cells, after 4-day induction of senescence, cells were washed twice with PBS to remove DTX in the medium to avoid its possible influence on the results. K1836 was thereafter added for 72 h with repeated supplementation every 24 h as post-DTX. In some experiments, K1836 was added 6 h before DTX as pre-DTX. Detailed analyses describing the phenotype of the DTX-induced senescent murine and human cells have been previously published (7,22,23).

### Flow cytometry

The proportion of live, apoptotic or necrotic cells were assessed using propidium iodide (PI) and Annexin V staining. Cells were harvested and centrifuged (4°C, 10 min, 180 × g); the pellet was washed in cold PBS, centrifuged (4°C, 10 min, 180 × g) and resuspended in Annexin V Binding Buffer (ApoFlowEx FITC Kit; cat. no. D7044, EXBIO Praha, a.s.). Annexin V-FITC and PI were added, mixed and incubated at room temperature in the dark for 15 min. After incubation, the cells were centrifuged (4°C, 10 min, 180 × g), resuspended in Annexin V Binding Buffer and then analysed using a BD FACS Symphony™ flow cytometer (BD Biosciences). Data were analysed using FlowJo 10 software (FlowJo LLC).

### MTT assay

Cells were seeded at a density of 5,000 cells/well in 96-well F microplates (Nunc; Thermo Fisher Scientific, Inc.). After 24 h of incubation at 37°C, the test compounds Stattic, K1823 and K1836 (dissolved in DMSO as 50 mM stock) were added to the cells in increasing concentrations 0–50 µM. MTT and DMF were added 24 and 30 h later, respectively. The absorbance was measured at 560 nm after 24 h of incubation at 37°C following DMF addition using a Thermo Scientific Multiskan EX (Thermo Fisher Scientific, Inc.).

### CellTiter-Glo luminescent cell viability assay

Cells were seeded in white, cell culture-treated, solid 384-well plates (Corning Inc.) at 1,000 cells/well in 20 µl media. Test compounds Stattic, K1823 and K1836 were diluted in DMSO and added to the cells using contact-free acoustic transfer using an Echo^®^ 655 Liquid Handler (Labcyte, Inc.; Beckman Coulter, Inc.) integrated in a fully automated robotic ACell HTS station (HighRes Biosolutions). Compounds were tested in the range 0.045–100 µM, in triplicate. Cells were incubated with the compounds for 72 h at 37°C. Cell viability was evaluated by assessing the level of intracellular ATP using a CellTiter-Glo^®^ (Promega Corporation) luminescent assay according to the manufacturer's protocol. The luminescent signal was quantified in an EnVision multimode plate reader (PerkinElmer, Inc.). Data were collected, normalized and processed using the ScreenX LIMS system (in-house developed proprietary system; http://www.openscreen.cz/en/screenx).

### Western blotting

Proliferating cells were seeded and incubated for 24 h, Stattic, K1823 and K1836 were added for 4 h and then cells were washed with cold PBS, harvested and lysed with 95°C heated modified Laemmli sample lysis buffer (1% SDS, 60 mM Tris-HCl, 10% glycerol and 0.4% β-mercaptoethanol in distilled water). Senescent cells were induced by DTX as aforementioned, washed with PBS and incubated in fresh medium with Stattic, K1823 and K1836 for 4 h; the lysates were then prepared as aforementioned for proliferating cells. Cell lysates were then heated at 95°C for 5 min and sonicated (3×30 sec at 3 µm amplitude and 40 kHz with 30 sec cooling intervals) using a Diagenode Bioruptor 300 (Diagenode SA). Before separation by SDS-PAGE on 10% gels, 0.01% bromophenol blue was added to the lysates. Equal amounts of protein (35 µg) were loaded per lane. After separation, proteins were electro-transferred onto a nitrocellulose membrane (Bio-Rad Laboratories, Inc.) using semi-dry transfer and were detected using primary antibodies at 4°C for 24 h, followed by incubation with a horseradish peroxidase-conjugated secondary antibody at room temperature for 1 h. GAPDH was used as a loading control.

### Reverse transcription-quantitative PCR (RT-qPCR)

RNA samples from TC-1 and TRAMP-C2 cell lines were isolated using an RNeasy Plus mini kit (Qiagen Sciences, Inc.) according to the manufacturer's protocol. cDNA was synthesized with random hexamer primers using a High-Capacity cDNA RT kit (Applied Biosystems; Thermo Fisher Scientific, Inc.). The temperature protocol for RT was 42°C for 30 min, 99°C for 5 min and 10°C for 5 min. qPCR was performed using an LC480II system (Roche Applied Science) with the SYBR™ Select Master mix (Applied Biosystems; Thermo Fisher Scientific, Inc.). The samples underwent a denaturation step (95°C for 6 min), followed by 42 amplification cycles (95°C for 30 sec, 60°C for 50 sec and 72°C for 70 sec), a melting step (95°C for 1 min, 65°C for 1 min and 95°C continuous acquisition) and cooling (37°C for 1 min). The relative quantity of cDNA was quantified using the 2^−ΔΔCq^ method (27). The sequence of the primers used were as follows: β-actin forward (F), 5′-CATTGCTGACAGGATGCAGAAGG-3′ and reverse (R), 5′-TGCTGGAAGGTGGACAGTGAGG-3′; IL-6 F, 5′-GGCCTTCCCTACTTCACAAG-3′ and R, 5′-ATTTCCACGATTTCCCAGAG-3′; GROα F, 5′-ACCCAAACCGAAGTCATAGC-3′ and R, 5′-TCTCCGTTACTTGGGGACAC-3′; and MCP-1 F, 5′-GAAGGAATGGGTCCAGACAT-3′ and R, 5′-ACGGGTCAACTTCACATTCA-3′, all primers were purchased from East Port Praha s.r.o. The final concentration of the primers used was 1 µM. Fold changes in transcript levels were calculated relative to β-actin, which was used as the endogenous reference gene control. The relative expression in the control group was normalized to 1. All samples were run in triplicate.

### ELISA

The protein levels of murine GROα were assessed in the supernatants of proliferating and senescent TC-1 and TRAMP-C2 cells using high-sensitivity ELISA kits (cat. no. DY453; R&D Systems, Inc.). After 4-day induction of senescence, cells were washed twice with PBS to remove DTX in the medium to avoid its possible influence on the results. K1836 was thereafter added for 72 h with repeated supplementation every 24 h. After cell treatment at 37°C, the medium was changed and the cells were cultivated for another 48 h in fresh medium. Experiments were performed according to the manufacturer's protocol and absorbance was quantified using a Thermo Scientific Multiskan EX (Thermo Fisher Scientific, Inc.).

### Cytokine cytometric bead array (CBA)

The levels of selected inflammatory factors (IL-6, IL-10, MCP-1, IFN-γ, TNF-α and IL-12) secreted by treated and untreated tumour cells were evaluated in media samples using a Mouse Inflammation Kit (cat. no. 552364; BD Biosciences) according to the manufacturer's protocol. The samples were analysed using a BD FACS Symphony™ flow cytometer (BD Biosciences). Data were analysed using FlowJo 10 software (FlowJo LLC).

### Data processing and statistical analysis

Graphs were generated using GraphPad Prism 9 (version 9.4.0; GraphPad Software; Dotmatics). Statistical analyses of graphs were performed using one-way ANOVA followed by Dunnett's post hoc test.

## Results

### Comparison of the cytotoxic effects of STAT3 inhibitors on proliferating and senescent (DTX-induced) murine tumour cells TC-1 and TRAMP-C2

**Figure 2.**
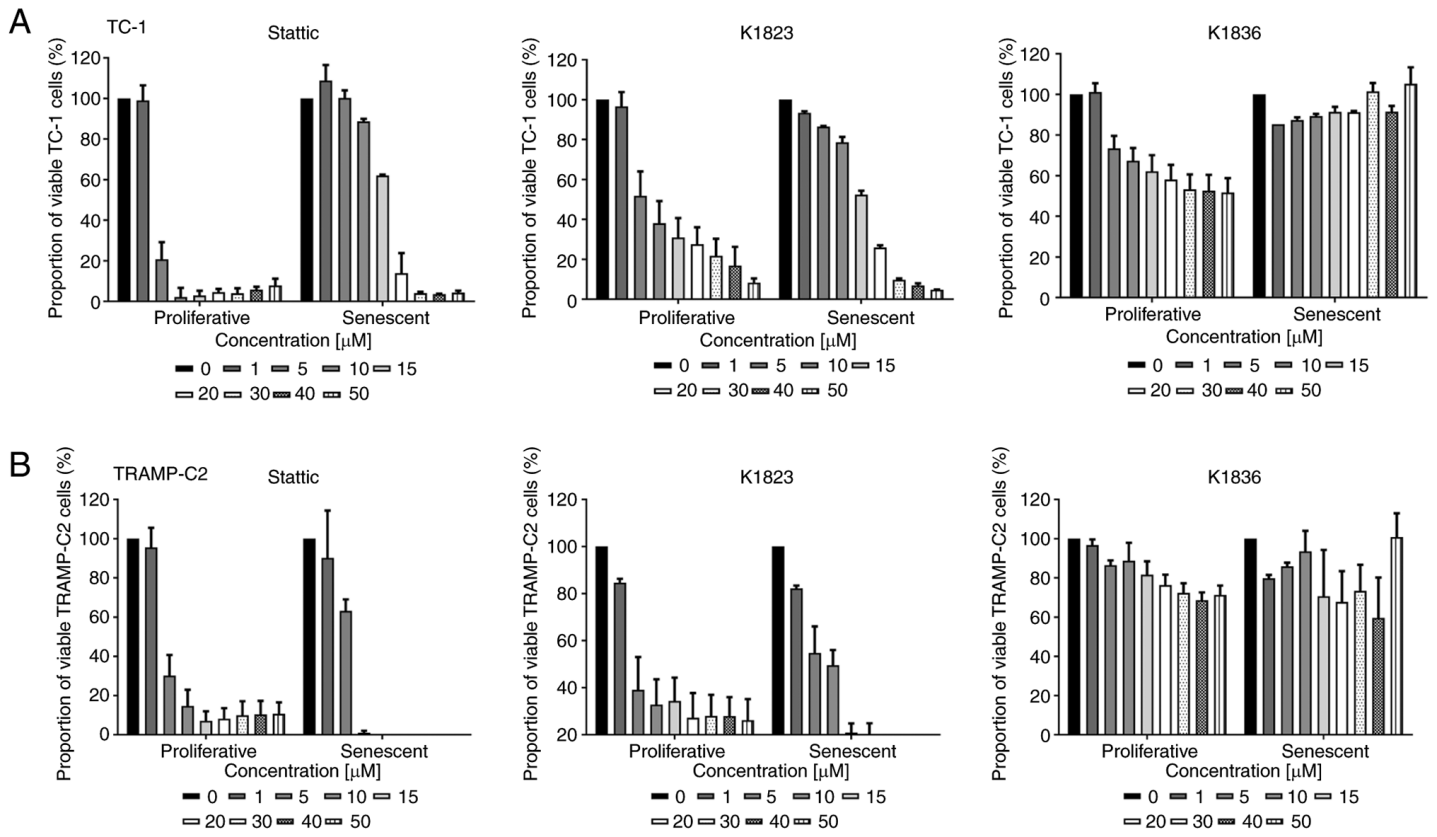
Cytotoxic effects of Stattic and novel STAT3 inhibitors on proliferating and senescent DTX-induced murine TC-1 and TRAMP-C2 cells. MTT assay of proliferating and senescent (A) TC-1 and (B) TRAMP-C2 cells treated with increasing concentrations (0–50 µM) of Stattic, K1823 and K1836. Data were normalized to untreated cells (in the case of proliferating cells) and DTX-treated cells (in the case of senescent cells). Data are presented as the mean ± SD (n≥3). Calculated IC_50_ values from the MTT assay are provided in Table I. DTX, docetaxel.

**Figure 3.**
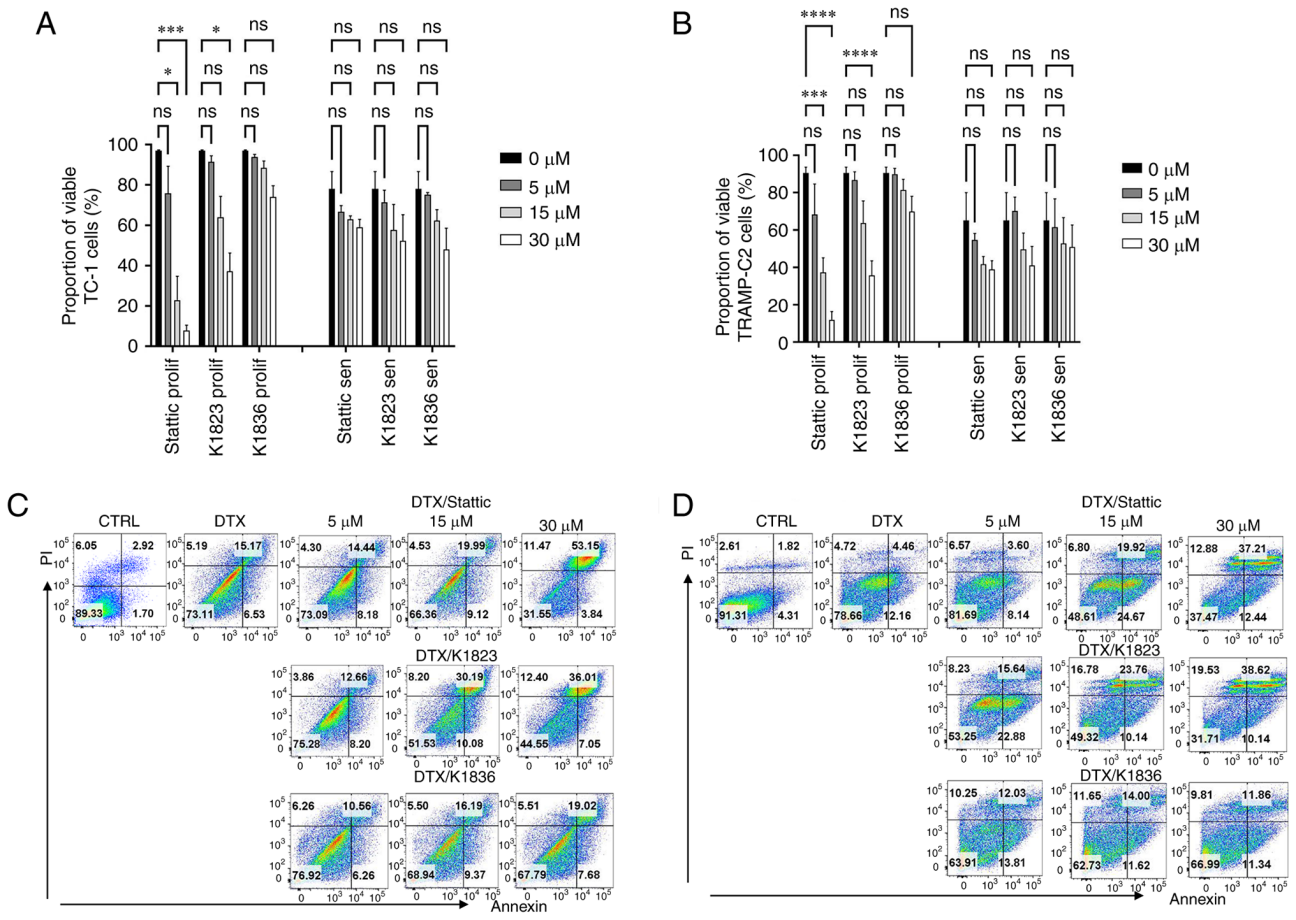
Cytotoxic effects of Stattic and novel STAT3 inhibitors on proliferating and senescent DTX-induced murine TC-1 and TRAMP-C2 cells. Viability of proliferating and senescent (A) TC-1 and (B) TRAMP-C2 cells treated with Stattic, K1823 and K1836 was assessed using apoptosis/necrosis flow cytometric analysis. Percentage of Annexin V-negative/PI-negative (i.e., viable) cells treated with three doses (5, 15 and 30) of Stattic, K1823 and K1836. Representative dot plots are presented for senescent (C) TC-1 and (D) TRAMP-C2 cells. Representative dot plots for proliferating TC-1 and TRAMP-C2 cells are shown in Fig. S5. *P<0.05, ***P<0.001 and ****P<0.0001 vs. CTRL. DTX, docetaxel; CTRL, untreated control; ns, not significant.

Two murine tumour cell lines, TC-1 and TRAMP-C2, which have constitutively elevated levels of activated pSTAT3, were used. Senescence was induced in these cell lines using 7.5 µM DTX as described previously (7,22). To compare the cytotoxic effects of commercially available STAT3 inhibitor Stattic and its two analogues, compound K1823 and newly synthesized K1836, proliferating and senescent TC-1 and TRAMP-C2 cells were treated with different concentrations of the tested compounds for 24 h and the cytotoxicity was assessed using the MTT assay. Both Stattic and K1823 demonstrated cytotoxic effects on both TC-1 and TRAMP-C2 proliferating cells. K1836 had a markedly lower cytotoxic effect compared with Stattic and K1823, as demonstrated by the IC_50_ values for particular inhibitors (Fig. 2; Table I). The MTT test generates a cell number estimation by measurement of their metabolic activity, so it does not discriminate between cell death and decreased cell proliferation. Therefore, flow cytometric analysis using PI and Annexin V staining was used to evaluate the early effects of the inhibitors on the cells. Proliferating and senescent cells were treated with 5, 15 and 30 µM Stattic, K1823 and K1836 for 24 h and the proportion of live cells was estimated as the percentage of Annexin V-negative/PI-negative cells (Figs. 3 and S5). Significant cytotoxic effects of both 30 µM Stattic and 30 µM K1823 compounds were demonstrated on proliferating cells, but not on senescent cells (Fig. 3A and B). However, cytotoxicity induced by K1836 was not statistically significant. The cytotoxic effects of Stattic and its two analogues, K1823 and K1836, following treatment of proliferating mouse TRAMP-C2 and human breast adenocarcinoma MDA-MB-231 cells for 24 or 72 h were assessed using the CellTiter-Glo luminescent cell viability assay. The results were similar to those using murine cell lines, as K1836 was the analogue with the lower cytotoxic activity. Longer incubation with the inhibitor decreased the IC_50_ in both mouse and human cell lines (Table II).

### Inhibitory effects of Stattic analogues on constitutive STAT3 phosphorylation in senescent cells

**Figure 4.**
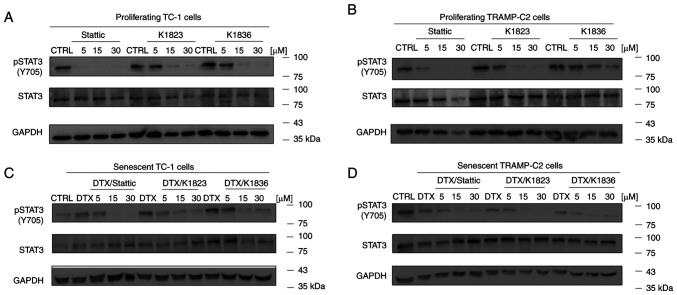
Inhibition of STAT3 phosphorylation by Stattic, K1823 and K1836 in (A) proliferating TC-1 cells, (B) proliferating TRAMP-C2 cells, senescent (C) TC-1 and (D) senescent TRAMP-C2 cells. Cells were treated with Stattic, K1823 and K1836 (5, 15 and 30 µM) for 4 h. pSTAT3 (Y705), total STAT3 and GAPDH protein expression levels were assessed with specific antibodies using western blotting. GAPDH was used as a loading control. p, phosphorylated; DTX, docetaxel; CTRL, untreated control.

The inhibitory effects of Stattic and the K1823 and K1836 analogues on STAT3 phosphorylation were evaluated using western blotting in proliferating TC-1 and TRAMP-C2 cells, both of which demonstrated constitutive phosphorylation of STAT3 (Fig. 4A and B). Cells were treated with 5, 15 and 30 µM Stattic, K1823 and K1836 for 4 h, and then the protein expression levels of pSTAT3 (Y705), as well as of total STAT3, were assessed. Furthermore, DTX-induced senescent cells (4 days after DTX treatment) were evaluated in the same way. Stattic and its analogues markedly inhibited Y705 STAT3 phosphorylation in both senescent TC-1 (Fig. 4C) and TRAMP-C2 (Fig. 4D) cells in a concentration-dependent manner, similar to the effect demonstrated in proliferating cells. The total STAT3 and pSTAT3 protein expression levels in the control TC-1 sample were constitutive but not stable, and continual changes in STAT3 protein expression levels in the TC-1 cell culture were noted in experiments, possibly due to oscillations in its expression.

### Effects of K1836 on the induction of senescence by DTX and the SASP of senescent cells

**Figure 5.**
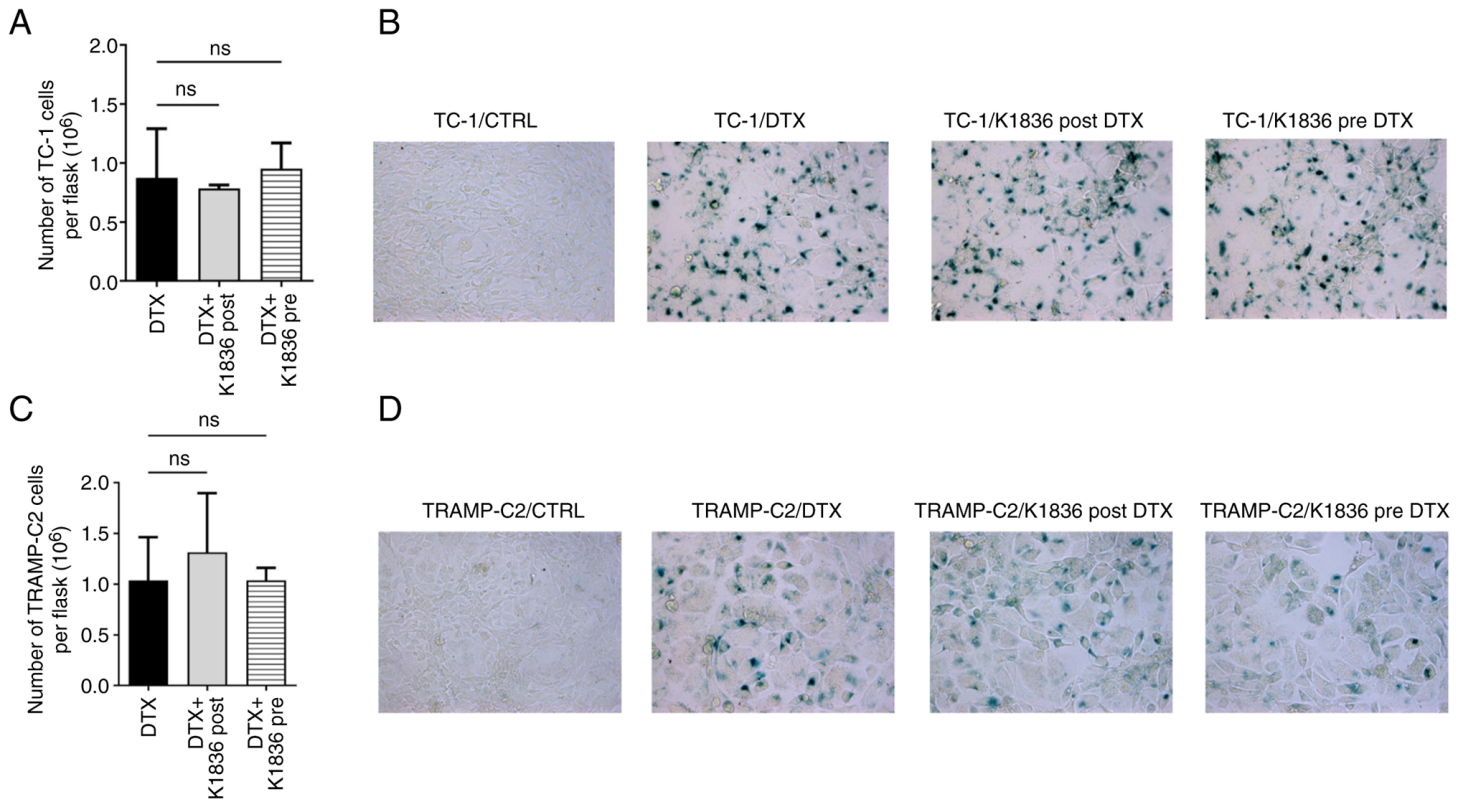
Effect of STAT3 inhibitor K1836 on senescence induction in TC-1 cells and TRAMP-C2 cells by DTX. (A) Proliferation was evaluated as cell count in flasks after pre-treatment of TC-1 cells with 15 µM K1836 (4 h before DTX supplementation) or as cell count in flasks after post-treatment cells with 15 µM K1836 (for 72 h after DTX supplementation). (B) The effect on induction of senescence in TC-1 cells was assessed by evaluation of the senescence-associated β-galactosidase activity. (C) Proliferation and (D) senescence induction were also assessed in TRAMP-C2 cells. DTX, docetaxel; CTRL, untreated control; ns, not significant.

How the STAT3 inhibitor K1836, which was only moderately cytotoxic but inhibited STAT3 phosphorylation in senescent cells, was able to affect the induction of senescence by DTX was evaluated. Both tumour cell lines were cultured with 15 µM K1836 for 72 h (with repeated supplementation every 24 h, as it was previously observed that STAT3 inhibition was not permanent and lasted for ~24 h; data not shown). K1836 was added to the cell culture medium either 4 h before adding 7.5 µM DTX (pre-treatment) or after 4 days of exposure to 7.5 µM DTX (post-treatment). There was no significant effect when K1836 was added either before or after DTX supplementation on the cell count or senescence of either the TC-1 (Fig. 5A and B) or TRAMP-C2 (Fig. 5C and D) tumour cell lines. The induction or presence of senescence upon DTX and inhibitors treatment was assessed using SA-β-gal activity in both TC-1 (Fig. 5B) and TRAMP-C2 (Fig. 5D) cell lines.

**Figure 6.**
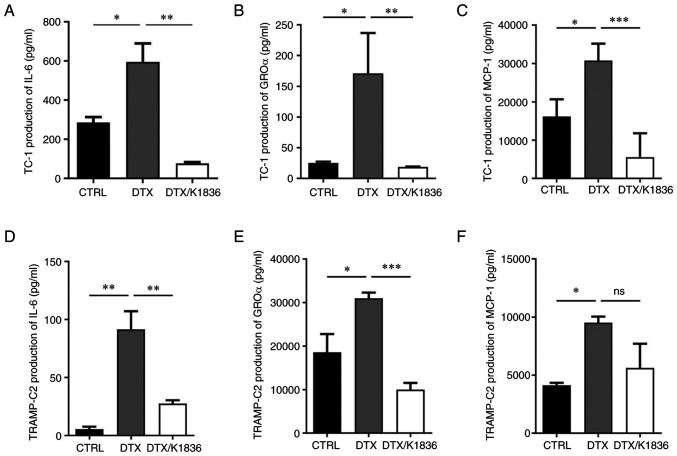
Secretion of GROα, IL-6 and MCP-1 by senescent TC-1 and TRAMP-C2 tumour cells after the treatment with 15 µM K1836. Protein levels of (A) IL-6 were assessed using ELISA, and (B) GROα and (C) MCP-1 were assessed for TC-1 cells using the cytometric bead array assay (representative dot plots shown in Fig. S6). Protein levels of (D) IL-6, (E) GROα and (F) MCP-1 were also determined for TRAMP-C2 cells. Supernatants were tested in triplicate, and the results from three independent experiments are presented as mean ± SD. *P<0.05, **P<0.01 and ***P<0.001 vs. DTX. GROα, growth-regulated oncogene α; IL, interleukin; MCP-1, monocyte chemoattractant protein-1; DTX, docetaxel.

**Figure 7.**
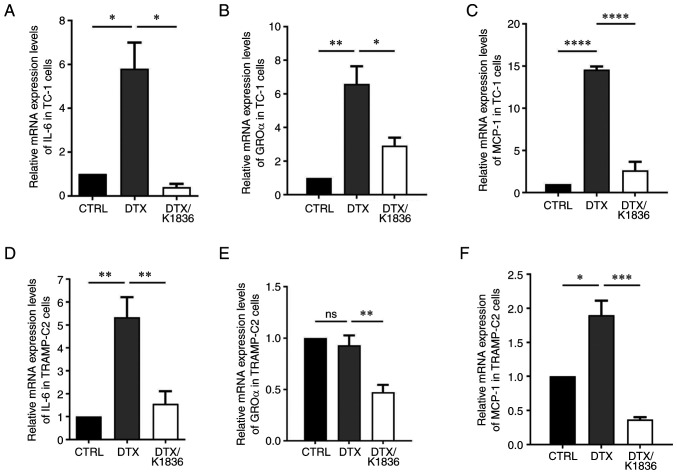
Relative mRNA expression levels of IL-6, GROα and MCP-1 in senescent TC-1 and TRAMP-C2 cells treated with DTX and DTX/K1836. Reverse transcription-quantitative PCR quantification of (A) IL-6, (B) GROα and (C) MCP-1 in control/untreated, DTX- and DTX/K1836-treated TC-1 and (D-F) TRAMP-C2 cells. β-actin was a housekeeping gene reference. Data from three independent experiments are presented as mean ± SD. Relative mRNA expression of genes are given as mRNA fold change in cells. *P<0.05, **P<0.01, ***P<0.001 and ****P<0.0001 vs. DTX. GROα, growth-regulated oncogene α; IL, interleukin; MCP-1, monocyte chemoattractant protein-1; DTX, docetaxel.

To analyse the effects of K1836 on the senescent cell phenotype in more detail, SASP modulation was evaluated. Changes in the expression and production of important SASP components upon K1836 treatment of senescent cells were assessed. ELISA and the CBA mouse inflammation kit were used to quantify the levels of selected SASP components (pro-inflammatory cytokines IL-6, GROα, MCP-1, IL-10, IFNγ and IL-12) in the supernatant from senescent cell cultures that were, after senescence induction, cultured for 72 h with 15 µM K1836 and then fresh medium for 48 h, before supernatant collection. Furthermore, the expression of the respective genes was assessed using RT-qPCR in corresponding cell samples. The secretion of IL-6 (CBA; Figs. 6B and E, and S6), GROα (ELISA; Figs. 6A and D, and S6), and MCP-1 (CBA; Figs. 6C and F and S6) was markedly increased in both TC-1 and TRAMP-C2 senescent cells compared with the control and were markedly reduced after the treatment of the senescent cells with K1836. The CBA analysis also demonstrated no changes in IFN-γ, TNF-α, IL-10 and IL-12 levels (data not shown). Corresponding significant reductions in IL-6, GROα, and MCP-1 mRNA expression levels were demonstrated in both TC-1 and TRAMP-C2 senescent cells following treatment with K1836 (Fig. 7).

## Discussion

In the present study, the effects of pSTAT3 Stattic and Stattic-derived small molecule inhibitors designed to target the STAT3 SH2 domain were evaluated. The results demonstrated that both proliferating and DTX-induced senescent cells of two murine cell lines were sensitive to these inhibitors in terms of their cytotoxicity and STAT3 Y705 phosphorylation; however, the cytotoxic effects on senescent cells appeared to be weaker. This agreed with the previous report that senescent cells can be more resistant to cytotoxic agents (28); therefore, it was not surprising that they were also less sensitive to the treatments compared with the proliferating cells. As these data were obtained using murine cell lines and there might be differences between murine and human cell lines, as well as between distinct cell types, the cytotoxic/cytostatic effects of the tested compounds were also evaluated using a human cell line.

Stattic was originally described as a specific pSTAT3 inhibitor, and its capability to bind the STAT3 SH2 domain, and effectively block its phosphorylation, dimerization and nuclear translocation, has been documented (21). Further studies have challenged this specificity (29), and a recent report suggested that the cytotoxic and pSTAT3 inhibitory effects of Stattic could be split, so its effects on cells cannot be attributed solely to its pSTAT3 inhibitory capacity (30). Although the present study did not perform mechanistic studies, the present results did indicate that K1836 was less toxic than the original compound but retained its capability to block STAT3 phosphorylation, which supported the aforementioned report. These features could also make K1836, or its possible derivatives, novel and helpful tools for the investigation of the STAT3 signalling pathway.

Aberrant activation of the STAT3 signalling pathway has been linked to tumour cell proliferation and tumourigenesis. However, activation of the STAT3 signalling pathway has also been reported to be associated with cellular senescence development and maintenance, and its activator, IL-6, has been described as a principal component of the paracrine or autocrine loops inducing senescence (31). However, there is a controversy on this point as several other studies reported that inhibition, not activation, of the IL-6/STAT3 signalling pathway induced cellular senescence in tumour cells. Therefore, theoretically, the impacts of the inhibitors on senescent cells could be distinct from those on proliferating cells, and it is interesting to see whether and how the pSTAT3 inhibitor could influence senescence development. The present study demonstrated, using two cell lines, that K1836 did not interfere with the development of DTX-mediated senescence. These data suggested that STAT3 activation, at least in the tested cell lines, was not crucial for the senescence development and maintenance.

However, K1836 treatment inhibited critical components of SASP, including IL-6, GROα and MCP-1 production, which could theoretically modify the tumour microenvironment and interactions of tumour cells with the immune system. The STAT3-induced production of cytokines/chemokines contributes to the pro-tumourigenic and inflammatory microenvironment, which is important for developing age-related diseases. It could therefore be hypothesised that the therapeutic effects mediated by the STAT3 inhibitors could be exerted not only by their cytotoxicity, but also by their capacity to change the cytokine milieu and the tissue microenvironment more generally. However, *in vivo* experiments evaluating possible therapeutic effects of the tested compounds and their capabilities to modify the tumour microenvironment have not yet been performed. The structure of the inhibitors may also require improvement from a pharmacological point of view before they can be tested in preclinical or clinical studies.

Collectively, chemotherapy-induced cellular senescence as a response to chemotherapeutic treatments has been considered as a positive event preventing carcinogenesis. Unfortunately, cellular senescence can serve an important role in chemotherapy resistance (32) and the creation of a pro-tumourigenic microenvironment accelerating tumour growth (7). Therefore, it is of particular importance to ensure that potentially cytotoxic or inhibitory agents, such as the tested STAT3 inhibitors, can target chemotherapy-induced senescent cells and that the selected compound, K1836, was able to modify the SASP by blocking secretion of pro-tumourigenic cytokines, including IL-6. The results of the present study suggested that optimized pSTAT3 small molecule inhibitors may be able to serve as anti-tumour, and maybe also senolytic/senomorphic agents.

## Supplementary Material

Supporting Data

## Figures and Tables

**Table I.** Comparison of the cytotoxic effects of STAT3 inhibitors on proliferative and senescent (DTX-induced) murine tumour cells TC-1 and TRAMP-C2.

	IC_50_			
	
Treatment	Proliferating TC-1 cells, µM	Senescent TC-1 cells, µM	Proliferating TRAMP-C2 cells, µM	Senescent TRAMP-C2 cells, µM
Stattic	3.46	15.80	3.92	5.38
K1823	3.58	14.94	5.77	6.27
K1836	49.47	N.D.	>50	N.D.

IC_50_ values were calculated from MTT tests performed using Stattic, K1823 and K1836. N.D., not defined.

**Table II.** Comparison of the cytotoxic effects of STAT3 inhibitors on proliferative mouse TRAMP-C2 and human MDA-MB-231 cells.

	IC_50_			
	
Treatment	TRAMP-C2-24 h, µM	TRAMP-C2-72 h, µM	MDA-MB-231-24 h, µM	MDA-MB-231-72 h, µM
Stattic	4.66	1.27	18.74	1.26
K1823	6.57	3.40	15.90	2.25
K1836	14.09	4.49	N.D.	2.83

IC_50_ values were determined using the CellTiter-Glo luminescent cell viability assay performed using Stattic, K1823 and K1836. N.D., not defined.

## Data Availability

The datasets used and/or analysed during the current study are available from the corresponding author on reasonable request.
