# Supplementary material for: STAT3 inhibitor Stattic and its analogues inhibit STAT3 phosphorylation and modulate cytokine secretion in senescent tumour cells
Source: Mol Med Rep. 2023 Feb 22;27(4):81. doi: 10.3892/mmr.2023.12968 (PMC10018236; doi:10.3892/mmr.2023.12968)

Figure S1. ^{1}H NMR spectrum of compound K1823.

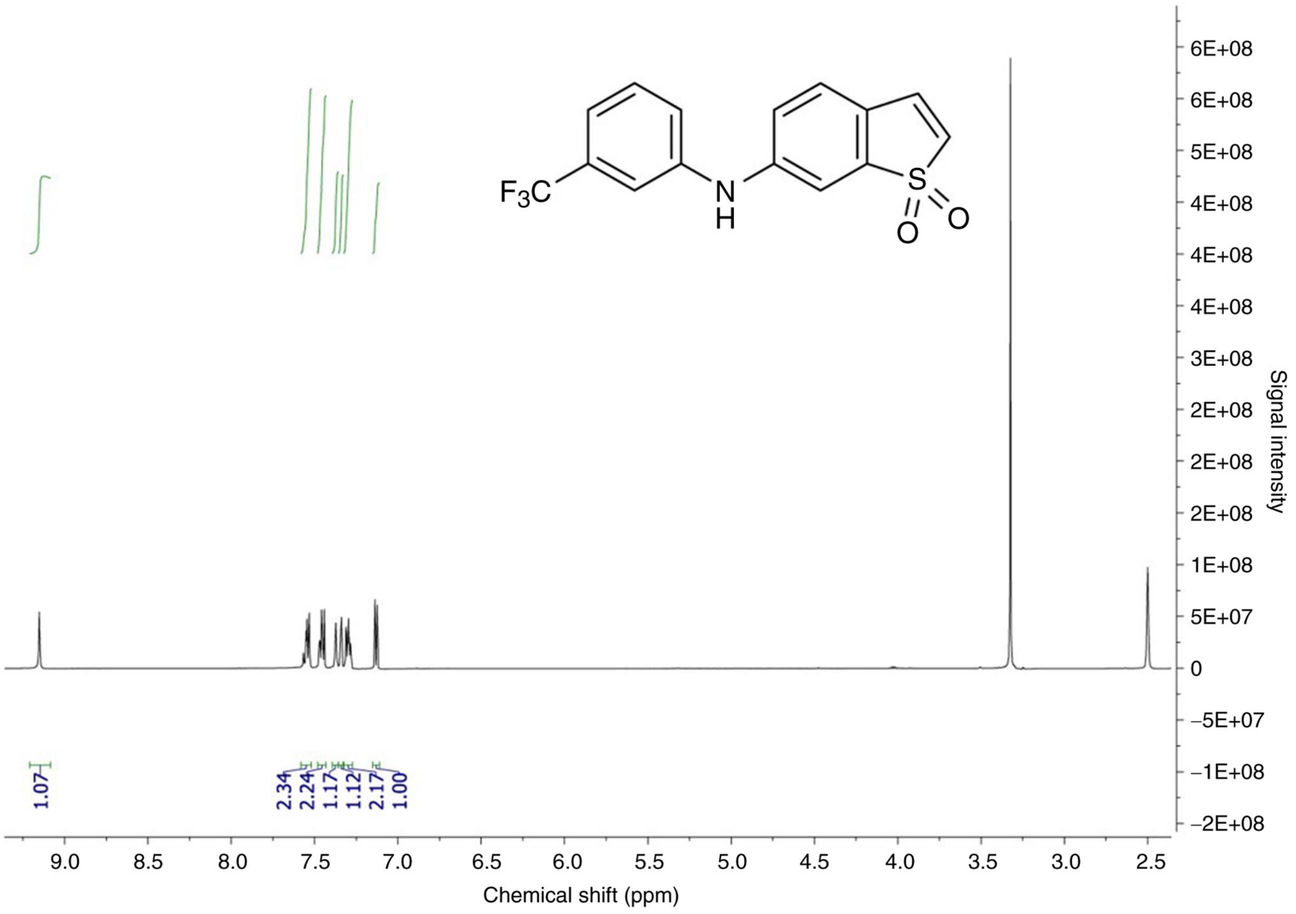

Figure S2. ^{13}C NMR spectrum of compound K1823.

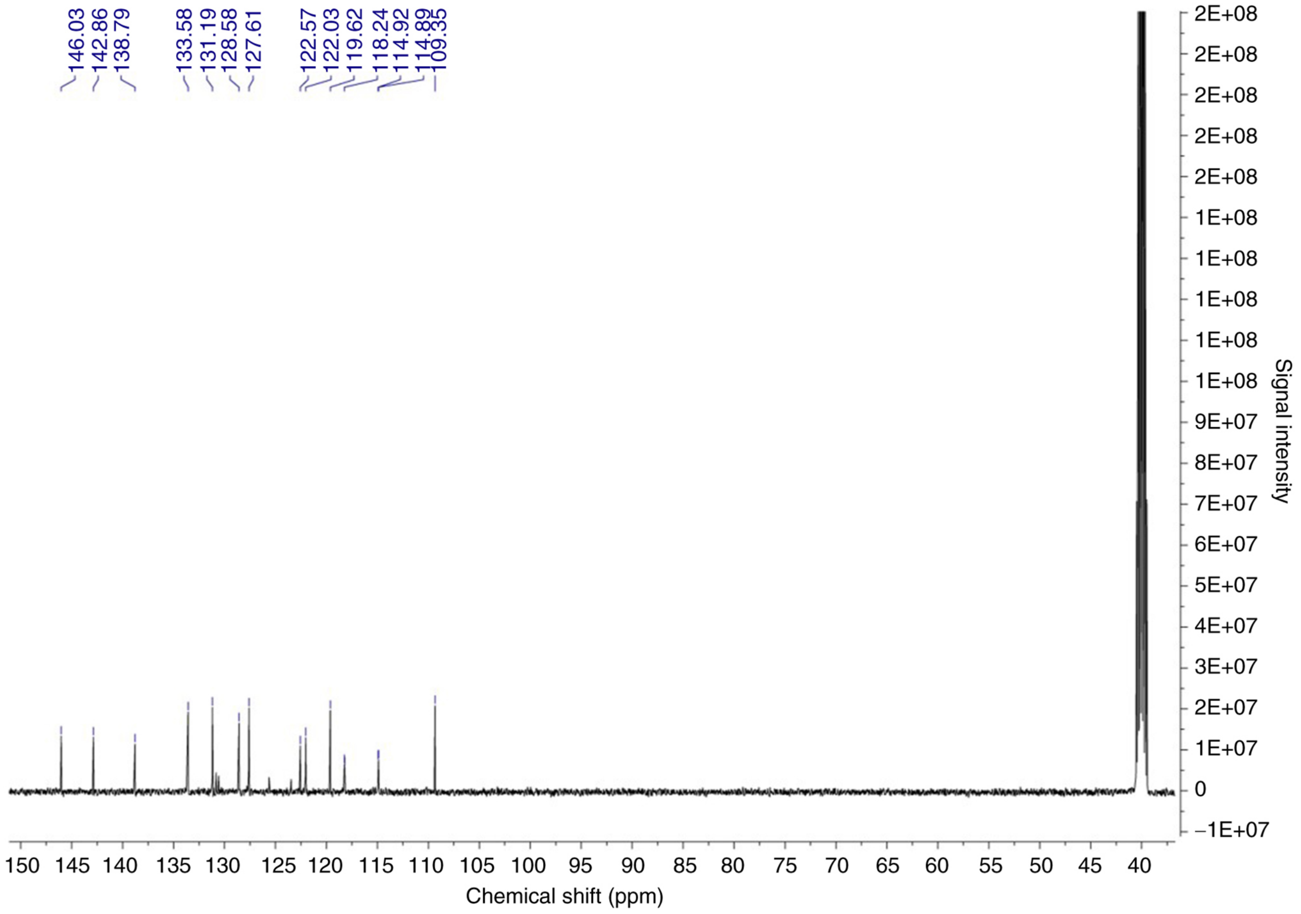

Figure S3. ^{1}H NMR spectrum of compound K1836.

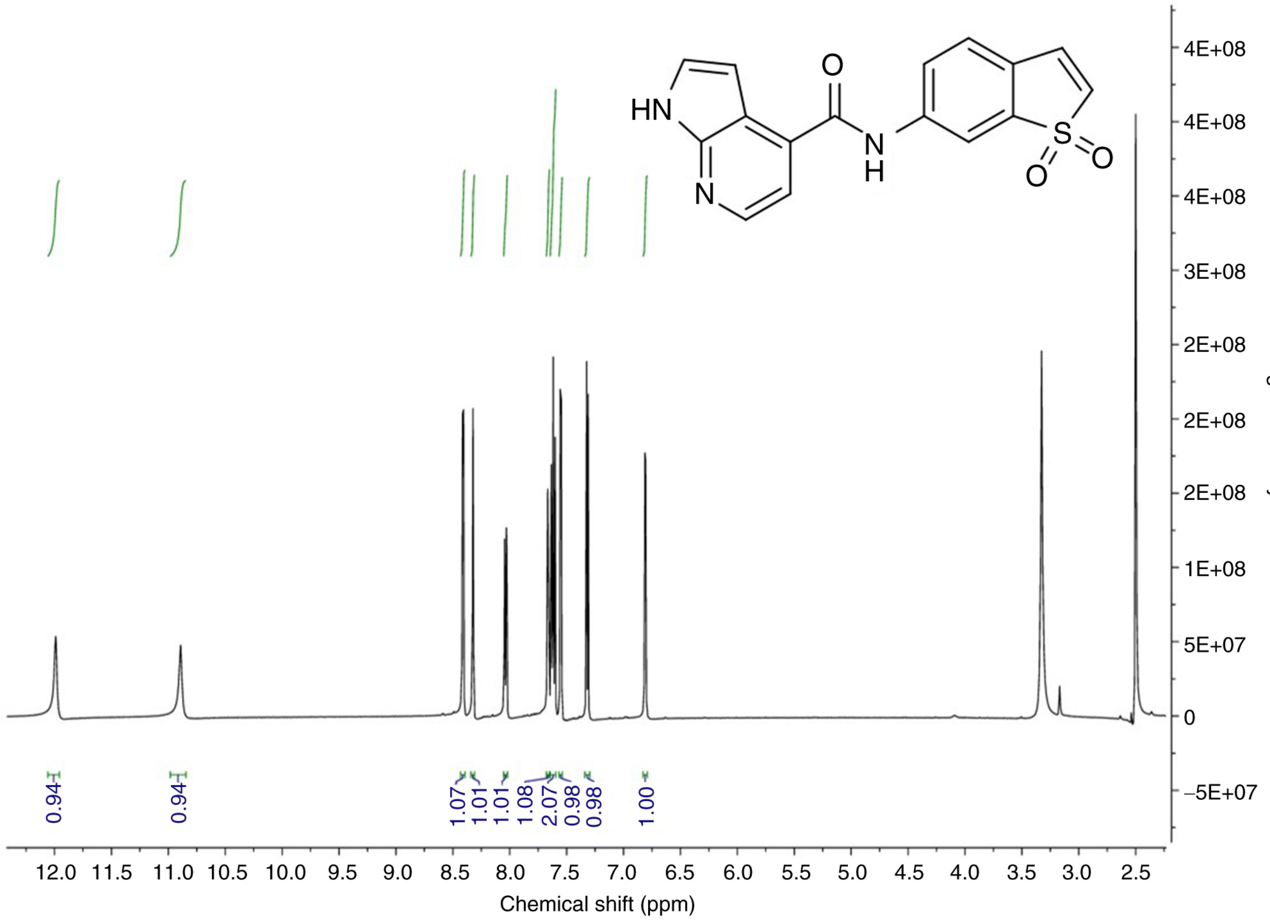

Figure S4. ^{13}C NMR spectrum of compound K1836.

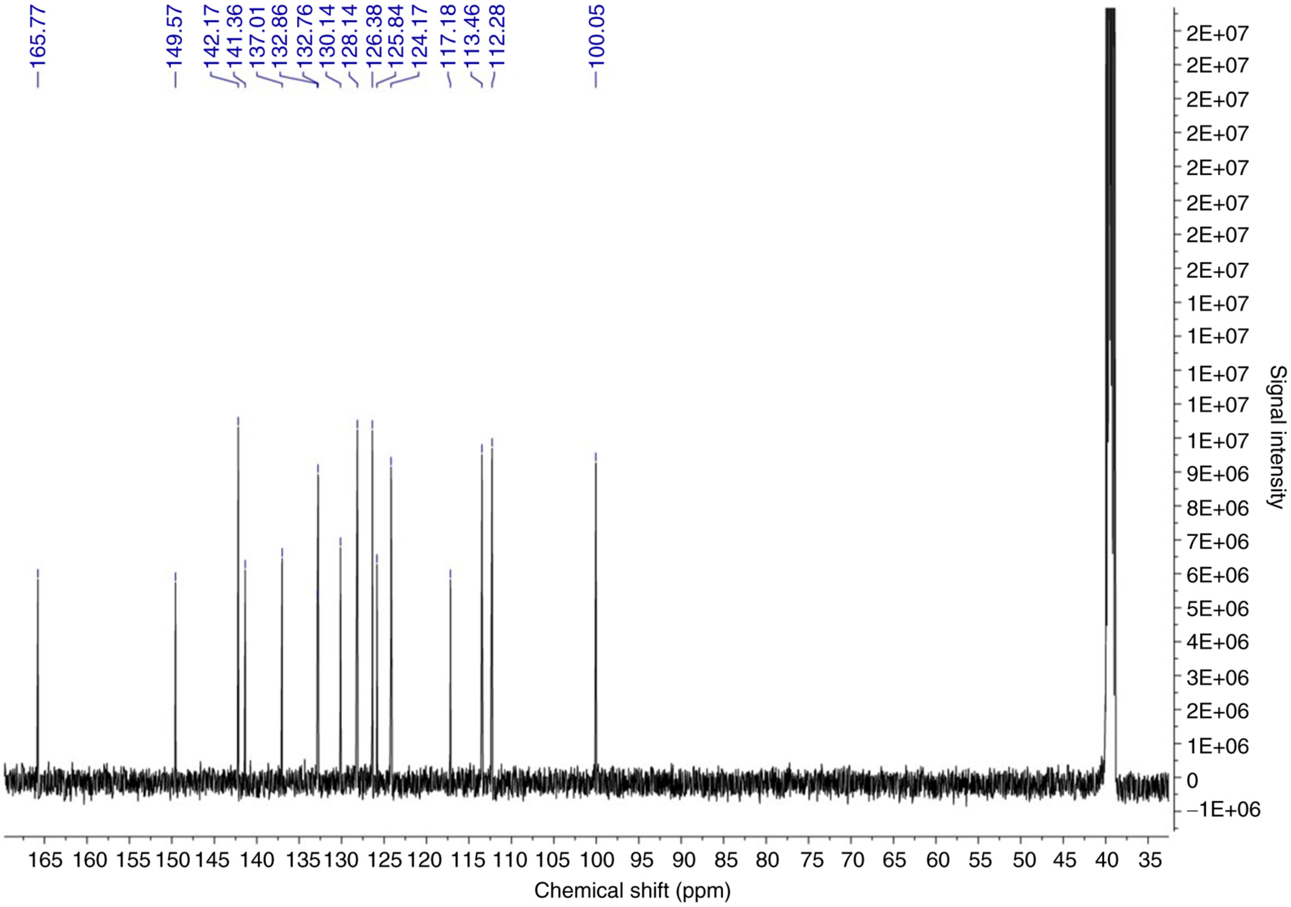

Figure S5. Cytotoxic effects of Stattic and novel STAT3 inhibitors on proliferating murine TC-1 and TRAMP-C2 cells. Representative dot plots for proliferating TC-1 and TRAMP-C2 cells.

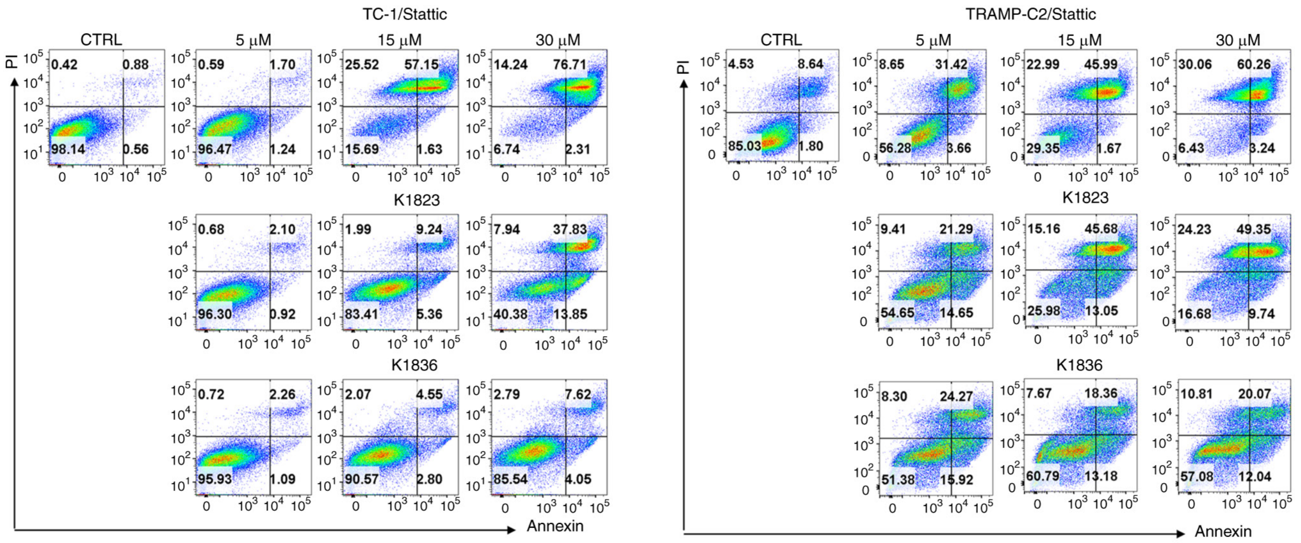

Figure S6. Secretion of IL-6 and MCP-1 by senescent TC-1 and TRAMP-C2 tumour cells after the treatment with 15 μM K1836. Protein expression levels of IL-6 and MCP-1 were assessed using the CBA assay. Representative dot plots for TC-1 and TRAMP-C2 cells.

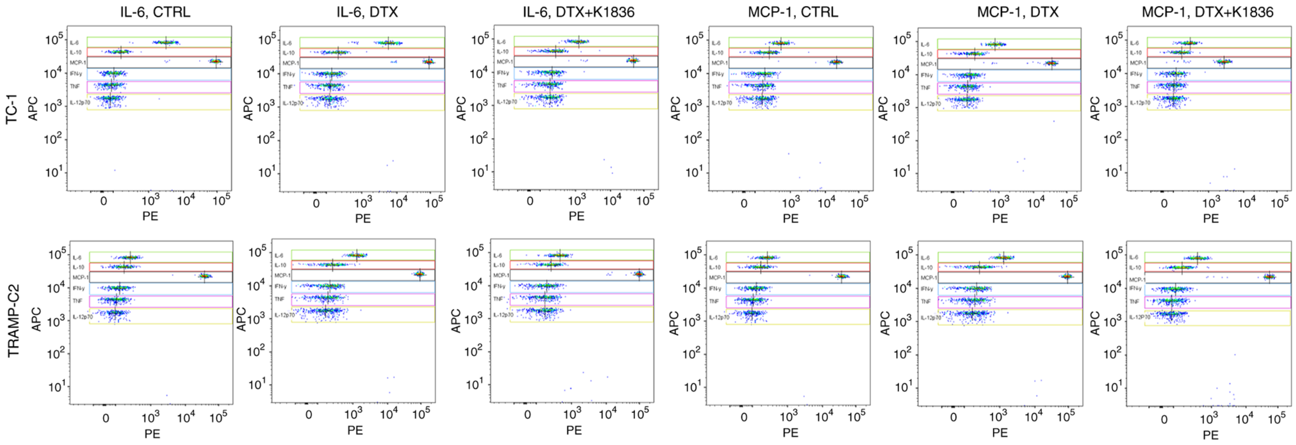

Supplement: Supporting Data [file Supplementary_Data.pdf]
